# Spectral domain optical coherence tomography predates fluorescein angiography in diagnosing central serous chorioretinopathy

**DOI:** 10.4103/0301-4738.60084

**Published:** 2010

**Authors:** Vishali Gupta, Amod Gupta, Pawan Gupta

**Affiliations:** Department of Ophthalmology, Advanced Eye Centre, Post Graduate Institute of Medical Education and Research, Chandigarh, India

Dear Editor,

Central serous chorioretinopathy (CSCR) is characterized by the development of serous retinal detachment in the macula and is mainly diagnosed by fluorescein angiography that shows one or multiple areas of leakage from the retinal pigment epithelium (RPE) seen as “expanding dot” or “smoke stack” sign.[1,2] The RPE defect corresponding to the dominant hyperfluorescence in the fluorescein angiogram, is believed to be the cause for serous leakage of fluid into the subretinal space and is seen as a pigment epithelium detachment (PED) on time domain Stratus Optical coherence tomography (OCT).[3] We present a case of CSCR where the spectral domain high-definition (HD) Cirrus OCT (Carl Zeiss Meditec) could diagnose subclinical CSCR before the development of the expanding dot sign on fluorescein angiography.

**Figure 1:**
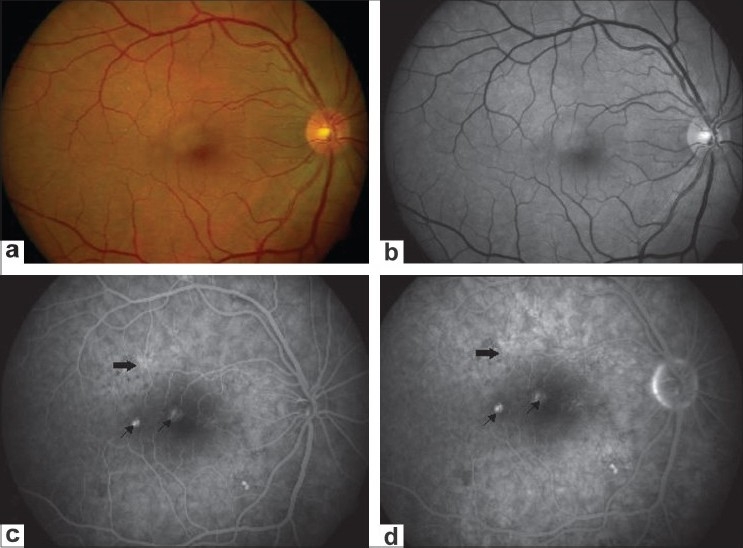
(a and b) Fundus and red-free photograph of the right eye showing mottled appearance superotemporal to the fovea. (c and d) Angiogram during transit and late phase showing hyperfluorescence suggestive of pigment epithelium detachment (arrows). Additionally, an area of punctate hyperfluorescence (solid arrow) is shown with an absence of smoke stack or subretinal fluid

**Figure 2:**
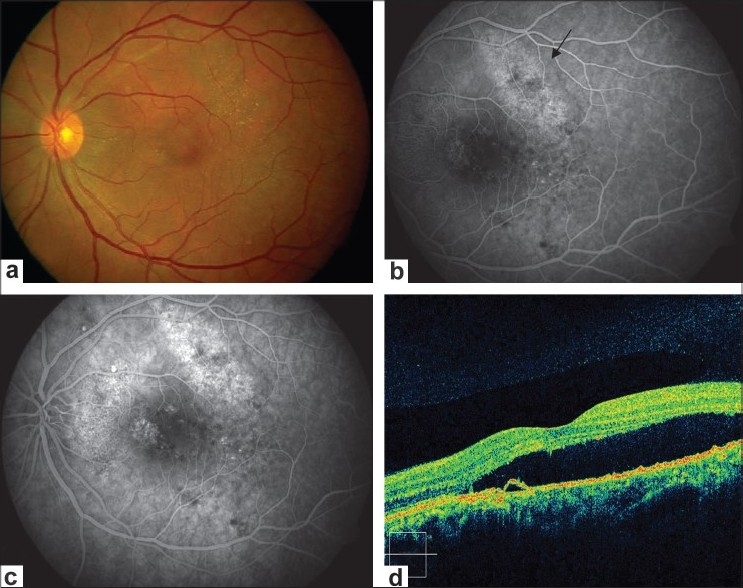
(a and b) Fundus photograph and angiogram of left eye showing pigmentry changes and yellowish precipitate, mottled appearance supero-temporal to fovea showing hyperfluorescence (arrows). (c) Additionally, showing multiple areas of pinpoint hyperfluorescence (d). OCT of left eye showing pigment epithelium detachment and subretinal fluid

**Figure 3:**
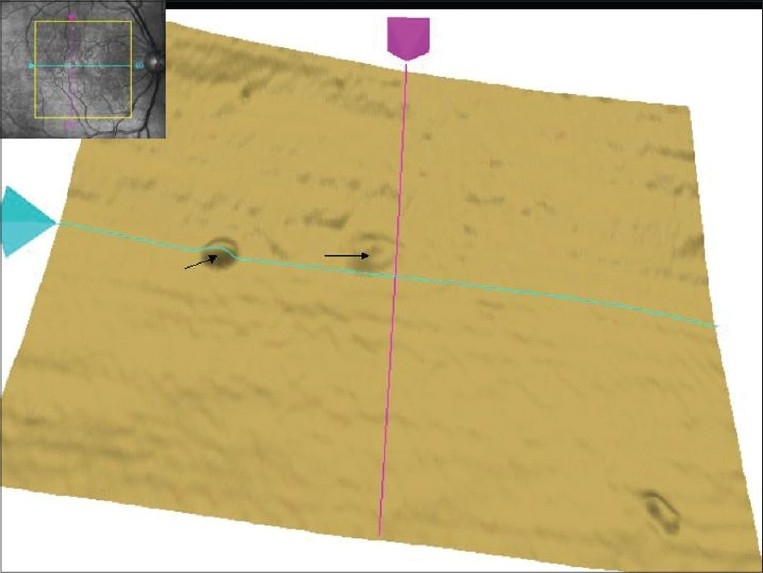
Single layer retinal pigment epithelium scan of right eye on Macular Cube 512 × 128 Combo shows pigment epithelium detachment s (arrows). In addition, the superior part of retinal pigment epithelium layer shows uneven surface with bumps. The inset shows fundus image with scan cube overlay

**Figure 4:**
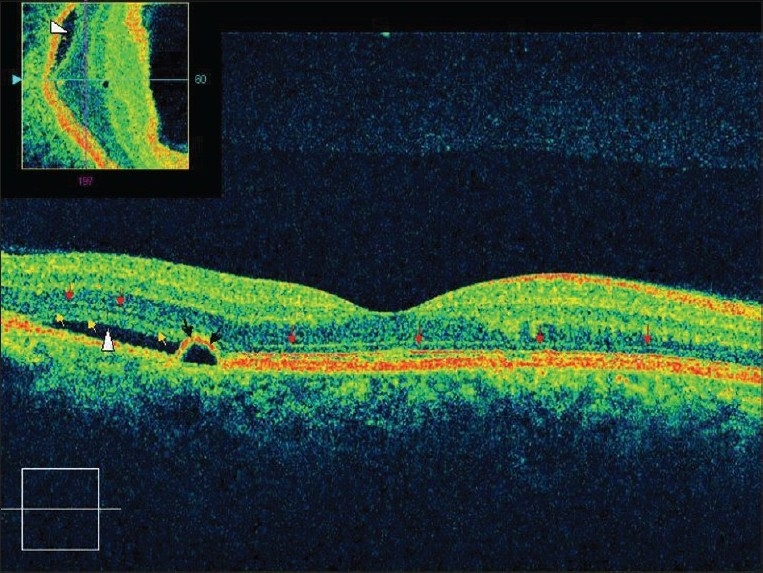
Raster Line scan of right eye passing above the fovea shows pigment epithelium detachment (black arrows), with subretinal fluid (arrowhead). Red arrows demarcate external limiting membrane while yellow arrows shows outer photoreceptor layer. Inset shows OCT slice showing the presence of subretinal fluid (solid arrow)

**Figure 5:**
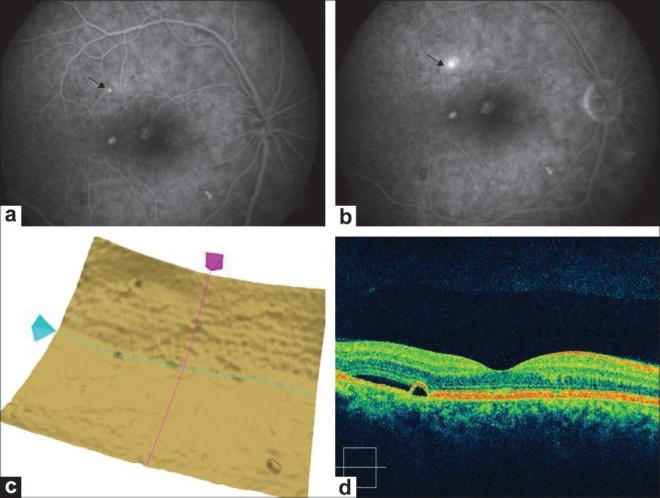
(a and b) Same eye(right eye) 3 months later shows expanding dot sign on fluorescein angiography (arrows). (c). Single layer RPE scan shows more uneven and bumpy surface compared to previous scan in Figure 2. (d). Raster Line scan right eye shows pigment epithelium detachment with subretinal fluid

A 40-year-old man was seen with complaints of blurred vision in his left eye with a visual acuity of 20/30 and was diagnosed to be suffering from CSCR in this eye. His right eye was asymptomatic with a visual acuity of 20/20. On fluorescein angiography, multiple areas of hyperfluorescence were seen that were diagnosed as PED. No expanding dot sign was seen at this stage in the right eye [Fig. 1] while the left eye showed multiple hyperfluroscence with late leakage [Fig. 2]. Analysis of the single-layer RPE showed elevations in the corresponding areas [Fig. 3]. A raster line scan of the right eye showed PED with serous retinal detachment [Fig. 4]. Similarly, the left eye showed PED with a serous retinal detachment [Fig. 2]. The patient was under regular follow-up for next few months. Three months later, the patient complained of blurred vision in his right eye (20/30) that now showed an expanding dot sign on fluorescein angiography [Fig. 5]. Repeat analysis of the single RPE layer now showed increased irregularity with bumps in the superior half [Fig. 5].

The diagnosis of CSCR is mainly clinical, aided by fluorescein angiography and OCT. Fluorescein angiography classically shows the leakage of dye from the choroid through a focal RPE defect and its pooling in the subretinal space in acute CSCR. OCT is traditionally used to quantify the amount and extent of the subretinal fluid, demonstrate thickening of the neural retina and is commonly used for monitoring during the follow-up and also for diagnosing the changes in the neurosensory retina that can cause permanent impairment in vision in such eyes.[3,4] Recently, 3D high-speed OCT has shown to facilitate the understanding of pathophysiologic changes in CSCR.[5] However, this case illustrates that spectral domain HD OCT could pick up the presence of the subretinal fluid and RPE changes even before they could manifest angiographically in CSCR. This indeed may be a better tool for monitoring the asymptomatic eye for the detection of early changes and may obviate the need for fluorescein angiography in selected cases. However, this is only a case report and a larger study is required.
